# The proton-sensing receptors TDAG8 and GPR4 are differentially expressed in human and mouse oligodendrocytes: Exploring their role in neuroinflammation and multiple sclerosis

**DOI:** 10.1371/journal.pone.0283060

**Published:** 2024-03-25

**Authors:** Fionä Caratis, Mikołaj Opiełka, Martin Hausmann, Maria Velasco-Estevez, Bartłomiej Rojek, Cheryl de Vallière, Klaus Seuwen, Gerhard Rogler, Bartosz Karaszewski, Aleksandra Rutkowska

**Affiliations:** 1 Brain Diseases Centre, Medical University of Gdansk, Gdansk, Poland; 2 Department of Anatomy and Neurobiology, Medical University of Gdansk, Gdansk, Poland; 3 Department of Gastroenterology and Hepatology, University Hospital Zurich, University of Zurich, Zurich, Switzerland; 4 H12O-CNIO Hematological Malignancies Group, Clinical Research Unit, Centro Nacional de Investigaciones Oncologicas (CNIO), Madrid, Spain; 5 Department of Adult Neurology, Medical University of Gdansk & University Clinical Centre, Gdansk, Poland; Anadolu University: Anadolu Universitesi, TURKEY

## Abstract

Acidosis is one of the hallmarks of demyelinating central nervous system (CNS) lesions in multiple sclerosis (MS). The response to acidic pH is primarily mediated by a family of G protein-coupled proton-sensing receptors: OGR1, GPR4 and TDAG8. These receptors are inactive at alkaline pH, reaching maximal activation at acidic pH. Genome-wide association studies have identified a locus within the TDAG8 gene associated with several autoimmune diseases, including MS. Accordingly, we here found that expression of *TDAG8*, as opposed to *GPR4* or *OGR1*, is upregulated in MS plaques. This led us to investigate the expression of TDAG8 in oligodendrocytes using mouse and human *in vitro* and *in vivo* models. We observed significant upregulation of TDAG8 in human MO3.13 oligodendrocytes during maturation and in response to acidic conditions. However, its deficiency did not impact normal myelination in the mouse CNS, and its expression remained unaltered under demyelinating conditions in mouse organotypic cerebellar slices. Notably, our data revealed no expression of TDAG8 in primary mouse oligodendrocyte progenitor cells (OPCs), in contrast to its expression in primary human OPCs. Our investigations have revealed substantial species differences in the expression of proton-sensing receptors in oligodendrocytes, highlighting the limitations of the employed experimental models in fully elucidating the role of TDAG8 in myelination and oligodendrocyte biology. Consequently, the study does not furnish robust evidence for the role of TDAG8 in such processes. Nonetheless, our findings tentatively point towards a potential association between TDAG8 and myelination processes in humans, hinting at a potential link between TDAG8 and the pathophysiology of MS and warrants further research.

## Introduction

To maintain pH homeostasis, cells are required to sense acidic changes in their extracellular environment and respond accordingly. Three G protein-coupled receptors (GPCRs), the ovarian cancer GPCR 1 (OGR1, GPR68), GPCR 4 (GPR4) and T cell death-associated gene 8 (TDAG8, GPR65) have been shown to sense extracellular protons and stimulate differing signalling pathways [1, 2]. The receptors are activated by acidic extracellular pH, through the protonation of several histidine residues located on the extracellular surface of the receptor [2]. At pH 7.6–7.8, the receptors are almost silent, and at pH 6.8–6.6, they are maximally activated [2].

Upon extracellular acidification, which is frequently observed in inflammation, proton activation of TDAG8 has been linked with anti-inflammatory roles in various disease models and in cells including inhibition of granulocyte, macrophage and monocyte inflammatory processes [3, 4]. In an earlier study, we examined the role of TDAG8 in inflammation in the experimental colitis, dextran sodium sulfate (DSS), model. We reported that TDAG8-deficiency leads to increased macrophage and neutrophil infiltration, and increased expression of pro-inflammatory mediators in both, the acute and chronic DSS models of colitis. Further, we examined acidosis-activated and TDAG8-mediated pathways in peritoneal macrophages by RNA-sequencing (RNA-seq). We found that activation of TDAG8 by low pH in peritoneal macrophages modulated the expression of genes involved in the immune response [5]. Additionally, as was demonstrated by us and others, TDAG8 can promote changes in cell adhesion and contractility, gene expression, cell division and proliferation [6].

Neuroinflammation, neurodegeneration, ischemic stroke and ageing are associated with acidosis in the central nervous system (CNS) [7–9]. In mice subjected to the middle cerebral artery occlusion (MCAO), the pH measured in the infarct region decreases to 6.5 compared to the ipsilateral hemisphere [10]. In Alzheimer’s disease, a neurodegenerative disease of the CNS, lower pH was found in the cerebrospinal fluid (CSF) and post-mortem brains [7]. Similarly, in multiple sclerosis (MS), a chronic autoimmune and inflammatory disease of the CNS, increased lactate levels were reported in the affected brain tissue [8]. Also in animals, a decreased pH was demonstrated in the spinal cords of mice in the experimental autoimmune encephalomyelitis (EAE) model of MS [11]. In the same mouse model, but in a different study, CNS acidosis was shown to be associated with demyelination and oligodendrocyte injury [12].

Despite the identification of TDAG8 as a risk gene for MS in high-powered genome-wide association studies (GWAS) [13, 14], limited research has been reported in this area. Only a handful of studies investigated the expression and signalling of TDAG8 in the CNS to date. For instance, expression of TDAG8 was reported in circumventricular organs in mouse microglia [15]. In this study, TDAG8-mediated signalling was involved in CO_2_-evoked freezing and sympathetic reactions in a mouse model of panic disorder. The TDAG8-deficient mice exhibited reduced activation of microglial cells and IL1β release in the subfornical area [15]. Additionally, it was demonstrated that the IL1β release is severely attenuated in LPS-activated microglia in an acidic environment through a TDAG8-dependent mechanism [16]. The expression of TDAG8 in another CNS-specific cell type was investigated by Bortell and colleagues [17]. They observed that TDAG8 is one of the three strongest expressed genes in astrocytes after *in vitro* treatment with methamphetamine. Interestingly, the other two upregulated genes, MAP2K5 and CXCL5, similar to TDAG8, are also inflammation and immune regulators. In the CNS, TDAG8 was also found to be expressed in neurons and to be negatively involved in chronic inflammatory pain [18, 19].

As for the role of TDAG8 in the EAE model, a single-cell RNA-seq (scRNA-seq) study and computational analysis used to determine different cellular states of Th17 cells revealed that TDAG8 promotes Th17 cell pathogenicity in the EAE model [20] via the Th17 cell master transcription factor, Rorγt, which binds the promoter region of TDAG8 [21]. However, another study where the effects of TDAG8 deficiency in the EAE model [22] were studied, found that TDAG8 deficient mice exhibit an exacerbated course of EAE and that TDAG8 signalling in invariant NK T cells, but not in CD4^+^ T cells, was responsible for attenuating the autoimmune responses [22]. Strikingly, the immunoregulatory effects of TDGA8 depended mostly on signalling in invariant natural killer (iNK) cells, but not CD4+ T cells [22]. Yet another study reported protection from the EAE in mice with TDAG8-deficient T cells [20]. The involvement of TDAG8 signalling in psychosine-mediated oligodendrocyte death in Krabbe disease (globoid cell leukodystrophy), a genetic demyelinating disease caused by the accumulation of psychosine, was investigated by Giri et al. [23]. In this study, using the MO3.13 human oligodendrocytes, the authors showed that the toxic effects of psychosine (galactosylsphingosine) on oligodendrocytes are TDAG8-independent, also demonstrating that psychosine is not a TDAG8 ligand.

## Materials and methods

### Ethics statement

Written informed consent was obtained from all human participants in accordance with the Declaration of Helsinki, and the study was approved by the Independent Bioethics Committee For Scientific Research at Medical University of Gdańsk, Poland under the licence number: NKBBN/457/2019.

Animal experiments were approved by the Local Ethical Committee for Animal Experiments in Bydgoszcz, Poland under licence numbers 27/2019 and 38/2021 and the Veterinary Authority of the canton of Zurich, Switzerland (in-house registration number 100635).

### Human participants

Written informed consent was obtained from all patients following the Declaration of Helsinki, and the study was approved by the Independent Bioethics Committee For Scientific Research at the Medical University of Gdańsk, Poland under the license number: NKBBN/457/2019. CSF and whole blood samples were collected from MS patients presenting with an initially clinically isolated syndrome during a routine diagnostic procedure or non-MS patients (controls) suspected of disorders requiring a lumbar puncture for diagnosis. Immediately after collection the CSF was centrifuged, aliquoted, and frozen at -80°C. All *in vitro* experiments were performed with pooled samples of CSF.

### Human MS and healthy brains

Post-mortem frozen human brain sections from MS and control donors were obtained from the Rocky Mountain MS Center Tissue Bank (Englewood, CO, USA) after approval by the Medical University of Gdansk (Poland) bioethics committee (NKBBN/253/2018). Lightly frozen 1 cm by 1 cm fragments were dissected from the white matter (WM) and periventricular WM plaques. Corresponding areas of WM were dissected from control brains. The fragments were deep-frozen in liquid nitrogen, ground using mortar and pestle, and homogenized in Fenozol reagent (A&A Biotechnology, 203–50). Total RNA was isolated from approximately 200 mg of homogenized tissue using the Total RNA Mini kit (A&A Biotechnology, 031–100) in an RNAase-free environment according to the manufacturer’s instructions. RNA quantification and quality control were performed spectrophotometrically at 260 nm and 280 nm using a PerkinElmer VICTOR Nivo plate reader (Perkin Elmer, USA) equipped with a μDrop Plate (Thermo Scientific, N12391). Isolated total RNA was stored at -20°C until used.

### Animals

The lipopolysaccharide (LPS)-challenge model was approved by the Local Ethical Committee for Animal Experiments in Bydgoszcz, Poland under license numbers 27/2019 and 38/2021. The C57BL/6 male mice were housed in standard cages with an enriched environment under 12-hour day and night cycles. The air in the room was exchanged 15 times per hour, and temperature and humidity were maintained at 20–23°C and 50–60%, respectively. The animals had unrestricted access to food and water.

The TDAG8 (GPR65) knock-out (KO) C57BL/6 mouse strain was generated and purchased from Deltagen, Inc. (San Mateo, CA, USA). The KO and littermate control mouse breeding was approved by the Veterinary Authority of the canton of Zurich (in-house registration number 100635). All the animals were housed in a specific pathogen-free (SPF) facility. The animals were kept in type II long clear-transparent individually ventilated cages (IVCs, Allentown, New Jersey, USA). They were fed a pelleted and extruded mouse diet (R/M–H Extrudat, Ssniff Spezialdiäten, Soest, Germany) *ad libitum*. The light/dark cycle in the room was given through natural daylight. The temperature was set to 21 ± 1°C, with a relative humidity of 55 ± 5% and 75 complete changes of filtered air per hour.

### Mouse brain isolation and immunohistochemistry

After decapitation whole brains from 20 weeks old male TDAG8 KO and wild-type (WT) mice were collected, carefully separated into hemispheres, and snap-frozen in liquid nitrogen. The hemispheres used for RT-qPCR were ground using mortar and pestle, homogenized in Fenozol reagent (A&A Biotechnology, 203–50), and stored at -20°C. For immunohistochemistry, the hemispheres were embedded in the O.C.T. matrix (VWR, 00411243). The tissue O.C.T. blocks were sectioned into 10 μm sagittal slices and mounted onto microscope slides. The slices were fixed in 4% ice-cold paraformaldehyde for 30 min at room temperature (RT) and washed with PBS. To avoid unspecific binding the slices were incubated in a blocking solution (10% BSA, 0.5% Triton X, 1% NGS in PBS) for 24 hours (h). Then the primary antibody solution (2% BSA, and 0,1% Triton-X in PBS) was applied and slices were incubated overnight in a humid box. All primary antibodies used in this study are shown in S1 Table. The slides were washed 3x for 30 min in PBS and incubated in a secondary antibody solution containing 1:500 dilution of anti-rabbit Cy3-conjugated (Jackson ImmunoResearch, 111-165-144) and anti-mouse Cy-5 conjugated (Jackson ImmunoResearch, 515-605-003) antibodies for 24 h. After mounting on glass cover slides the slices were imaged with Zeiss LSM880 (Zeiss, Oberkochen, Germany) confocal microscope and analysed using Zeiss Zen 3.5 software.

### LPS challenge model

Male C57BL/6 wild-type mice were used in the model. The mice received intraperitoneal (i.p.) injections of 0.9% NaCl (vehicle) or 2 mg/kg LPS for 24 h. The mice were anaesthetized with isoflurane and perfused transcardially with NaCl. Whole brains were removed and the right hemisphere was snap-frozen in liquid nitrogen. The hemispheres were homogenized with a pestle and a mortar in liquid nitrogen. The resulting powder was then suspended in Fenozol reagent (A&A biotechnology, 203–50). Total RNA was isolated from approximately 50 mg of homogenized tissue using the Total RNA Mini kit (A&A Biotechnology, 031–100).

### Organotypic slice culture and human CSF treatments

Organotypic cerebellar slice cultures were prepared from brains isolated from postnatal 10 days old C57BL/6 mice as specified in the protocol published earlier [24–27]. After decapitation cerebellum was carefully separated from the hindbrain with a spatula and cut into 400 μm sagittal slices using McIlwain tissue chopper (Campden Instruments, TC752). The slices were transferred into a petri dish containing ice-cold Opti-MEM (Invitrogen, 11058021) and separated using sterile needles under a magnifying glass. Four to six slices per condition were inserted onto a cell culture insert (Millicell, PICMORG50) and cultured in a humidified incubator (35.5°C and 5% CO_2_) for 14 days in complete media consisting of 50% Opti-MEM, 25% HBSS, 25% horse serum, 1% glucose, 1% HEPES buffer, 1% GlutaMax and 1% pen/strep. For remyelination experiments, after 14 days in complete media, the slices were starved for 4 h in complete media without the horse serum and demyelinated with 0.4 mg/ml lysophosphatidylcholine (LPC) for 18 h, followed by recovery for 18–20 days in serum-free media (SFM) consisting of Neurobasal-A, B-27 supplement, HBSS, glucose, HEPES buffer, GlutaMax and pen/strep. Media change was performed three times per week. For CSF treatment experiments, after 14 days in complete media, the slices were starved for 4 h in complete media without the horse serum and then incubated in 10% pooled CSF from either control or MS patients in SFM media overnight. The slices from the remyelination and CSF treatment experiments were collected from the inserts with a tip and suspended in Fenozol reagent (A&A biotechnology, 203–50) and total RNA was isolated using the Total RNA Mini kit (A&A Biotechnology, 031–100).

### Primary mouse oligodendrocyte progenitor cell culture and differentiation

Primary OPCs were prepared from postnatal day 5 to 7 (P5/7) C57BL/6 mice. The procedure follows briefly Dincman and colleagues paper on OPC isolation (Dincman, 2012). The cortical tissue was placed in a petri dish already containing HBSS without calcium and magnesium. The tissue, without olfactory bulbs and meninges, was then processed for neural dissociation (130-092-628, Miltenyibiotec) using the gentleMACS^™^ dissociator (without heaters). Once the tissue was dissociated, it was then processed for selection of CD140a positive oligodendrocyte precursors (130-101-502, Miltenyibiotec). Resulting OPCs were then cultured on poly-d-lysine coated flasks for 6–10 days in OPC media (DMEM/F12, N2 supplement (1%), B27 supplement (2%), P/S (1%), BSA (0,01%), FGFb (40ng/ml), PDGF-AA (20ng/ml)) at 37°C and 5% CO2. Half media change was performed every other day. Once they reached confluency, cells were detached with accutase and plated on poly-d-lysine coated plates, 10^5 cells/ml. After 2–3 days, OPC media was replaced by differentiation media (DMEM/F12, N2 supplement (1%), B27 supplement (2%), P/S (1%), insulin (50ug/ml), triiodo-thyronine T3 (40ng/ml) for subsequent experiments.

#### Cell culture and differentiation

MO3.13 human oligodendrocytic hybrid cell line (RRID: CVCL_D357) was purchased from Tebu-Bio (2018, CLU301, batch 131117 P25) and cultured in high glucose DMEM (Sigma Aldrich, D5796), 10% FBS and 1% pen/strep. Cells were plated on 6-well cell culture-treated plates and differentiated using 100 nM phorbol 12-myristate 13-acetate (PMA) (Sigma-Aldrich, P1585) for 72 h in serum-free RPMI 1640 medium (SFM, Sigma Aldrich, R8758) supplemented with 2 mM Glutamax and 20 mM HEPES with pH adjusted to pH = 7.6 (high pH), pH = 7.25 (normal pH) or pH = 6.8 (low pH) [28]. The medium in each group was changed every 24 h. After treatments, MO3.13 cells were washed in PBS and scraped in 200 μl of Fenozol reagent (A&A biotechnology, 203–50). Total RNA was isolated using the Total RNA Mini kit (A&A Biotechnology, 031–100).

### pH shift experiments

The pH shift experiments and differentiation experiments were conducted in SFM supplemented with 2 mM Glutamax and 20 mM HEPES. The pH of the solutions was adjusted using calibrated pH meter (Mettler Toledo, Seven Compact S220) with 1M NaOH or 1M HCL. The initial pH of the media was adjusted to pH = 8.1 (high pH) or pH = 6.1 (low pH). The media were filtered and equilibrated in a 5% CO_2_ incubator for at least 72 h before experiments, and the pH was measured again. After equilibration, the final pHs were 7.6 (high pH) and 6.8 (low pH), measured at RT. The pH of the normal, unadjusted SFM was 7.2. Undifferentiated MO3.13 cells were plated on 6-well cell culture-treated plates and grown until reached 70% confluence. The cells were starved for 4 h in a serum-free high pH medium. The high pH medium was removed and the pH shift was performed by adding the high, normal, or low pH medium. The cells were cultured for up to 72 h depending on the experiment. After treatments, the cells were washed in PBS and scraped in 200 μl of Fenozol reagent (A&A biotechnology, 203–50). Total RNA was isolated using the Total RNA Mini kit (A&A Biotechnology, 031–100).

### pH shift experiments in inflammatory conditions

The MO3.13 oligodendrocytes were plated on 6-well cell culture-treated plates and cultured until approximately 70% confluency. The cells were starved for 4 h in serum-free high pH medium and treated with LPS (100 ng/ml) (Sigma Aldrich, L4391) in serum-free high, normal, or low pH medium for 2 h or 24 h. For the IL17/TNFα challenge experiments, the cells were starved in a serum-free high pH medium and then incubated overnight with a cocktail of 10 ng/ml TNFα (R&D Systems, 210-TA-100/CF) and 50 ng/ml IL-17A (R&D Systems, 317-ILB-050) recombinant cytokines. After treatments, MO3.13 cells were washed in PBS and scraped in 200 μl of Fenozol reagent (A&A biotechnology, 203–50). Total RNA was isolated using the Total RNA Mini kit (A&A Biotechnology, 031–100).

### Cell migration assay

Undifferentiated MO3.13 cells were starved for 2 h before the experiment in serum-free high pH medium. Migration assay was performed using 8.0 μm cell culture inserts (Corning, 353097) on 24-well plates. Each treatment condition was duplicated and each experiment was repeated at least three times. The cells were trypsinized, centrifuged, and resuspended in a high pH medium to obtain 800.000 cells/ml solution. Subsequently, 400 μl of high pH medium, low pH medium, or high pH medium supplemented with 0,01 μM 7-alpha,25-dihydroxycholesterol (7α,25OHC) (Sigma Aldrich, 64907-22-8) was added to the bottom of the chamber. Subsequently, 80 μl of the cell solution was added carefully into the insert top chamber. After 30 min at RT, the top chamber was topped up with 80 μl of high pH medium. The cells were left to migrate to the lower chamber for 24 h. Then the media was discarded from the chamber and the remaining cells were removed with a cotton swab. The transwells were immersed in 250 μL of crystal violet staining solution (Sigma Aldrich, CAS 548-62-9) for 10 min at RT. Then, they were carefully dipped in a beaker with distilled water and left to air dry. When dried, images were taken with a light microscope, and the transwells were incubated in 250 μL of extraction solution (Methanol, Cell Biolabs, CBA-100) for 20 min on a shaker. The solution was transferred to a 96-well plate to read absorbance at 590 nm with a VICTOR NivoTM plate reader (Perkin Elmer, USA).

### Real-Time Quantitative Polymerase Chain Reaction (RT-qPCR)

For reverse cDNA transcription, the Transcriba reverse transcription kit was used (A&A Biotechnology, 4000–100) following the manufacturer’s instructions. The RT-qPCR was performed using Sensitive RT HS-PCR Mix (A&A Biotechnology,2017–149 2000) on the Light Cycler 480 (Roche) under the following conditions: 120 s at 50°C, the 20s at 95°C then 40 cycles at 95°C for 3 s and 60°C for 30 s. Total RNA isolated from cerebellar slices, TDAG KO and control WT mice was reverse transcribed using the High-Capacity cDNA Reverse Transcription Kit (Applied Biosystems, 4368814). RT—qPCR was performed on the LightCycler 480 (Roche) using the TaqMan FAST Universal Mastermix (Applied Biosystems, 4304437). Cycling conditions were: 20 seconds (s) at 95°C, then 45 cycles at 95°C for 3 s, and 60°C for 30 s. The FAM dye-labelled TaqMan probes (Applied Biosystems) were used in all experiments and are listed in S2 Table. The relative mRNA expression was determined using the ΔΔCt method, calculated from absolute quantification after normalization to the endogenous housekeeping gene (β-actin).

### Cell immunohistochemistry

The MO3.13 oligodendrocytes were cultured on 8-well imaging slides (Millipore, PEZGS0816) covered with Poly-D-Lysine in SFM low pH, normal pH, or high pH medium for 24 h. The cells were washed once with cold PBS, fixed for 10 min in ice-cold 4% PFA, and permeabilized for 60 s in cold 100% methanol. Cells were incubated in blocking solution (0.5% NGS, 1% BSA, 0.1% tween-20 in PBS) for 60 min and then overnight in primary antibodies diluted in PBS containing 0.5% BSA and 0.05% tween-20 at 4°C. The cells were washed 3x with PBS and incubated for 1 h at RT in a secondary antibody solution containing 1:500 dilution of anti-rabbit Cy3-conjugated (Jackson ImmunoResearch, 111-165-144) and anti-mouse Cy-5 conjugated (Jackson ImmunoResearch, 515-605-003) antibodies. In the final step, the nuclei of the cells were counterstained with Hoechst dye (ThermoFisher, H1399), washed, and imaged with Zeiss LSM880 (Zeiss, Germany) confocal microscope and analysed using Zeiss Zen 3.5 software.

### Human OPC and microglia material

The cDNA for human OPCs was acquired from ScienCell (1604), and human microglia total RNA was procured from Sti (37089RNA). RNA extraction was carried out using the fenozol-based Total RNA Mini Plus kit (A&A Biotechnology, 036–100,) as per the manufacturer’s instructions. The quantity and quality of mRNA were assessed through spectrophotometric analysis using a plate reader (Biotek, Synergy). RNA concentration was determined based on the optical density at 260 nm, and the samples were subsequently standardized. Subsequently, cDNA synthesis was performed using the TranScriba Kit (A&A Biotechnology, 4000–100,) following the manufacturer’s protocols. RT-qPCR was conducted using TaqMan Master Mix (Thermo Fisher, 4444556,) on the LightCycler480 (Roche, Switzerland). FAM dye-labeled TaqMan probes from Applied Biosystems (CA, United States) were employed. To calculate relative mRNA expression, the ΔΔCt method was utilized, with normalization to a reference gene based on absolute quantification.

### Statistical analysis

All Statistical analyses were performed using *GraphPad Prism* statistical software (version 8.0 or higher, RRID: SCR_002798). Multiple comparisons between the experimental groups were made using one-way analysis of variance (ANOVA) followed by Sidak’s post-hoc tests for comparisons of differences between selected experimental groups, Dunnett’s post-hoc tests for comparisons of the control with other means and Tukey’s post-hoc test for comparisons of all means with every other mean. Multiple comparisons between the experimental groups and normalised control were made using one sample t-test. Comparisons between two groups were made using independent student *t*-tests. All data are presented as means ± standard error of the mean (SEM). Each experiment was independently repeated at least 3 times (N = 3). Throughout this article significant differences are indicated by asterisks: *p ≤ 0.05, **p ≤ 0.01, ***p ≤ 0.001, ****p ≤ 0.0001.

## Results

### TDAG8 is upregulated in MS plaques

**Fig 1:**
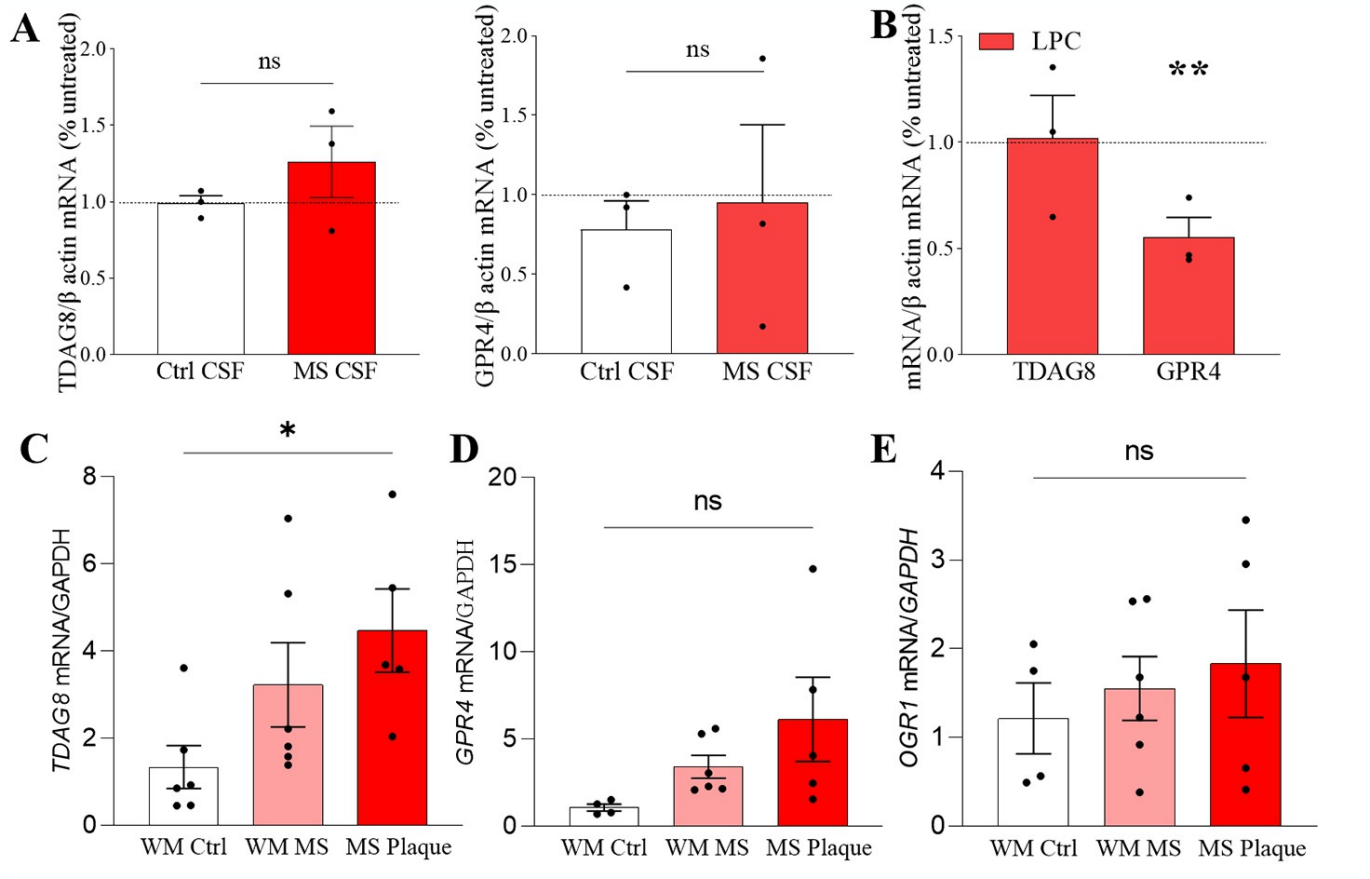
TDAG8 is upregulated in MS plaques. A. Overnight incubation of mouse organotypic cerebellar slices with the CSF from control or MS patients did not affect *tdag8* or gpr*4* expression. The dotted line indicates expression in untreated slices. One sample t-test, p>0.05, N = 3 independent experiments. B. The mRNA transcripts of *tdag8* were unchanged in LPC-demyelinated cerebellar slices. However, The mRNA levels of *gpr4* were significantly downregulated after LPC treatment (55% +/- 9% ctrl vs. LPC). Unpaired student t-tests, **p≤0.01, N = 3 independent experiments. The dotted line indicates expression in untreated slices. C. The mRNA levels of *TDAG8* in the normal-appearing WM in MS brains were upregulated 2.4-fold compared to non-MS controls and significantly upregulated 3.3-fold in MS plaques. D. and E. The mRNA transcripts of *GPR4* and *OGR1* were not differentially expressed in MS brains. One-way ANOVA and Tukey’s post-hoc tests, *p≤0.05, N = 5–6 human brains.

Neuroinflammation, neurodegeneration or ischemic stroke are associated with local tissue acidification [10, 29–32]. We therefore begun our study by examining the expression of TDAG8 in MS, for which *TDAG8* is a risk gene [13, 14]. We first treated mouse organotypic cerebellar slices with the CSF from MS patients or non-MS controls. The data showed no differences in *tdag8* or *gpr4* expression after treatment with either control or MS CSF (Fig 1A). However, when the slices were challenged with LPC, a demyelinating agent, the expression of *gpr4* significantly decreased during remyelination while *tdag8* transcripts remained at the same level as in the untreated slices (Fig 1B). Finally, we analysed the receptors’ expression in the WM in MS and non-MS human brains. Among the three proton-sensing receptors examined, only the mRNA transcripts of *TDAG8* were differentially expressed in MS plaques (Fig 1C–1E). The mRNA levels of *TDAG8* were already somewhat elevated in the normal-appearing WM in the MS brains compared to the matching WM areas in non-MS brains and significantly upregulated in the WM lesions (MS plaques) compared to the WM in the control brains.

#### Peripheral inflammation modulates the expression of *tdag8* and *gpr4* in the mouse brain

**Fig 2:**
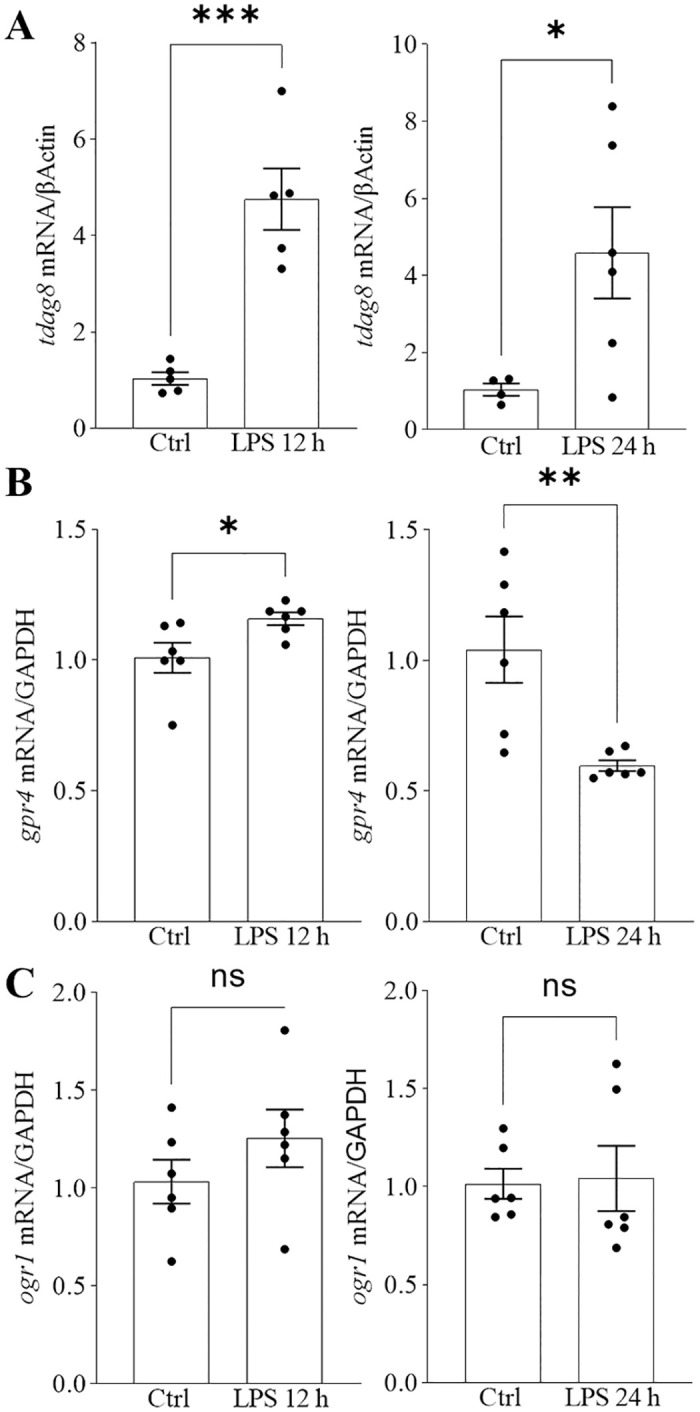
Peripheral inflammation modulates the expression of *tdag8* and *gpr4* in the mouse brain. A. I.P. injection of LPS induced *tdag8* expression in the mouse brain 4.6-fold 12 hours after injection and 4.4-fold 24 hours post-injection. B. *Gpr4* mRNA levels increased 1.1-fold 12 hours post LPS injection and decreased 0.6-fold 24 hours after injection. C. The expression of *ogr1* in the mouse brain was not affected by LPS. Student t-tests, *p≤0.05, **p≤0.01; ***p≤0.001, N = 5–6 mice per condition.

I.p. injections of LPS were shown to induce neuroinflammation and significant pH reduction in the mouse brain [30, 33]. TDAG8, is mainly expressed in the immune cells and has anti-inflammatory properties, while GPR4 is expressed in the immune cells and the CNS and has pro-inflammatory properties [34–38]. We therefore asked the question of whether TDAG8 is involved in inflammatory signalling in the brain. We began by inducing inflammation in cultured human MO3.13 oligodendrocytes during a pH shift with a cocktail of TNFα/IL17 cytokines, which were shown before to induce pro-inflammatory signalling and cytokine release in glial cells [33, 39]. Here, in the human MO3.13 oligodendrocytes, the overall *TDAG8* expression was downregulated after 18 hours of TNFα/IL17 treatment in high, normal and low pH with significance reached for cells grown in high and low pH (S1A Fig). We then tested the effects of bacterial LPS on *TDAG8* expression in these cells. The data showed that stimulation of MO3.13 oligodendrocytes with LPS significantly induces mRNA expression of *TDAG8* short- (2 h) and long-term (24 h) (S1B Fig). Subsequently, we investigated the dynamics of mRNA expression of TDAG8, GPR4 and OGR1 in mice subjected to LPS challenge. After administering either LPS or vehicle to mice, we examined the receptor expression in whole brain homogenates at 12 and 24 hours following injection. Consistently with our *in vitro* experiments using human MO3.13 oligodendrocytes, i.p. injection of LPS resulted in a substantial induction of *tdag8* expression in the mouse brain at both 12 and 24 hours (Fig 2A). While peripheral administration of LPS did not affect *ogr1* expression in the mouse brain (Fig 2C), the mRNA levels of *gpr4* exhibited differential modulation. There was an initial increase, observed at 12 hours post LPS injection, followed by a decrease at 24 hours post-injection (Fig 2B). As the data indicated opposing trends in the mRNA levels of *tdag8* (increasing) and *gpr4* (decreasing) 24 hours after LPS injection, we proceeded to investigate their expression in mice lacking *tdag8* (TDAG8 KO mice). The results revealed the expression of *ogr1* and *gpr4* were significantly upregulated in *tdag8*-deficient mice brains (S2 Fig).

#### TDAG8 deficiency does not affect normal myelination in the mouse brain

**Fig 3:**
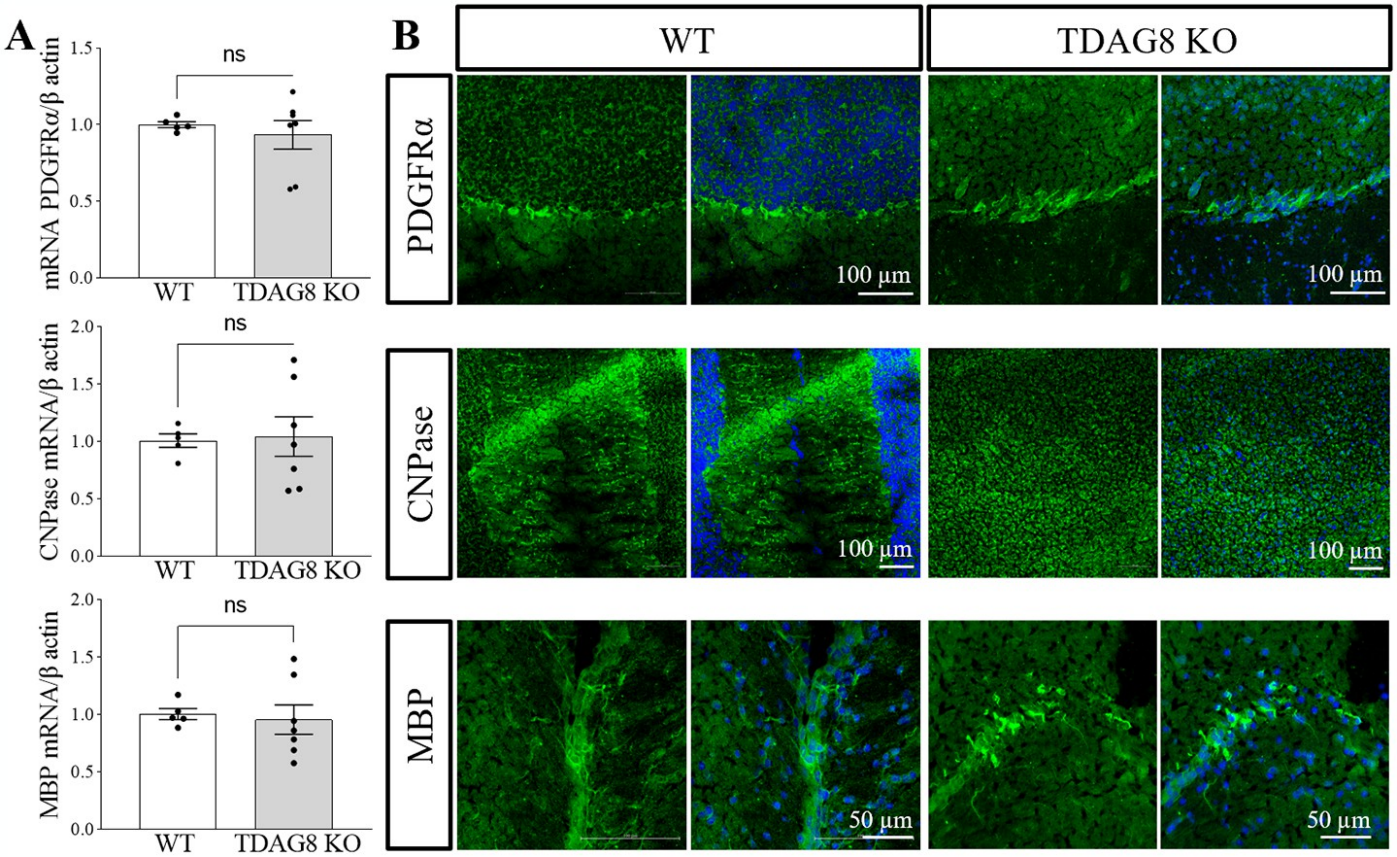
TDAG8 deficiency does not affect normal myelination in the mouse brain. A. The mRNA analysis of *pdgfrα*, *cnpase* and *mbp* expression in whole brain homogenates did not reveal differences between the TDAG8 KO and WT mice. Student t-tests, p>0.05, N = 5 WT, N = 7 TDAG8 KO mice. B. Representative images of immunohistochemically stained WT and TDAG8 KO mice brains (cerebellum) show no differences in myelination between the two genotypes.

Going back to investigating the potential role of TDAG8 in CNS myelination we examined if TDAG8 deficiency affects normal myelination in the mouse brain. We analysed OPC and oligodendrocyte markers at gene and protein levels in adult WT and TDAG8 KO mice. The data showed no differences between the WT and TDAG8 KO brains in the mRNA levels of the OPC marker PDGFRα, the early myelination marker CNPase, nor in the mature myelin marker MBP (Fig 3A). The immunohistochemical staining of the brain sections from the WT and TDAG8 KO mice also did not reveal qualitative differences in the PDGFRα, CNPase and MBP proteins (Fig 3B).

#### TDAG8 is not expressed in primary mouse OPCs

**Fig 4:**
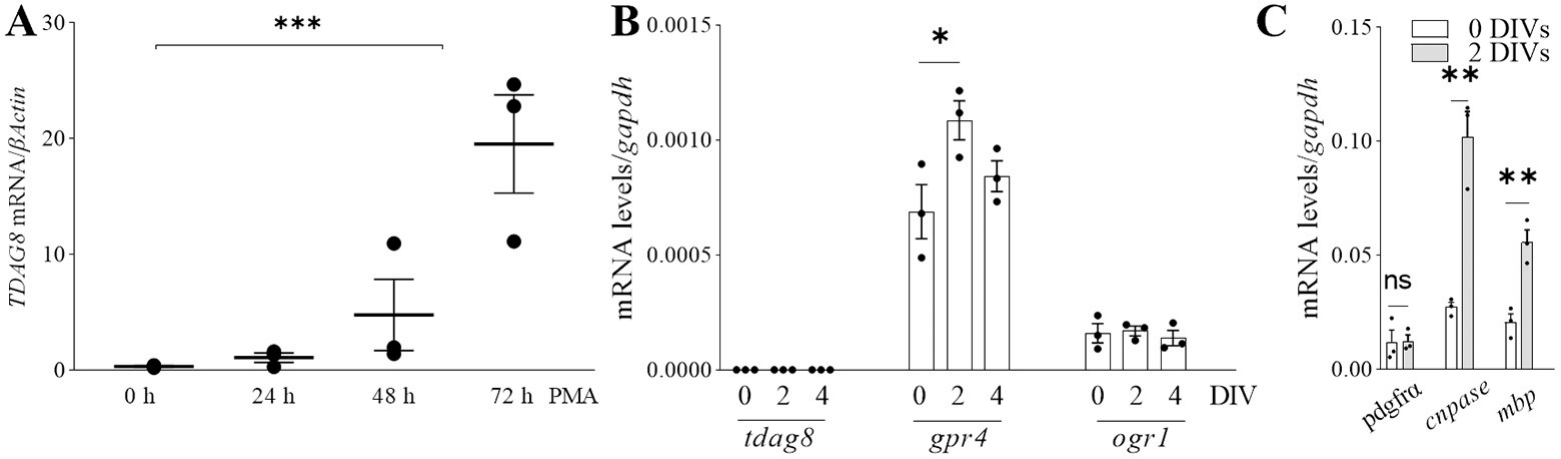
TDAG8 is not expressed in primary mouse OPCs. A. The mRNA expression of TDAG8 steadily increases in PMA-stimulated MO3.13 oligodendrocytes (60.38% +/- 13.11% 0 h vs. 72 h). One-way ANOVA and Dunnett’s post-hoc tests, ***p≤0.001, N = 3 independent experiments. B. Primary mouse OPCs do not express *tdag8* even after 4 DIV differentiation but *gpr4* is strongly expressed and its expression increases with differentiation. *Ogr1* is stably expressed in OPCs but its expression does not change with differentiation. One-way ANOVA and Dunnett’s post-hoc tests, *p≤0.05, N = 3 independent experiments. C. Increasing CNPase and MBP expression in primary mouse OPCs during 0–2 DIVs differentiation induced with T3. One-way ANOVA and Dunnett’s post-hoc tests, **p≤0.01, N = 3 independent experiments.

The lack of differences between the WT and TDAG8 KO brains in myelination state (Fig 3) and no changes in *tdag8* expression during remyelination in the brain organotypic slices (Fig 1B) prompted us to examine if TDAG8 is present in the mouse OPCs and oligodendrocytes. In the human cells, the data showed a steady increase in *TDAG8* expression during MO3.13 maturation with significant upregulation after 72 hours of stimulation with the maturation-inducing molecule in this cell line, PMA (Fig 4A) [28]. Conversely, *tdag8* was not expressed in undifferentiated and differentiated (up to 4 DIVs) primary mouse OPCs. In comparison, *gpr4 and ogr1* were expressed in mouse OPCs, with *gpr4* levels increasing with OPC maturation (Fig 4B and 4C).

#### TDAG8 is upregulated in human MO3.13 oligodendrocytes during maturation and under acidic conditions

**Fig 5:**
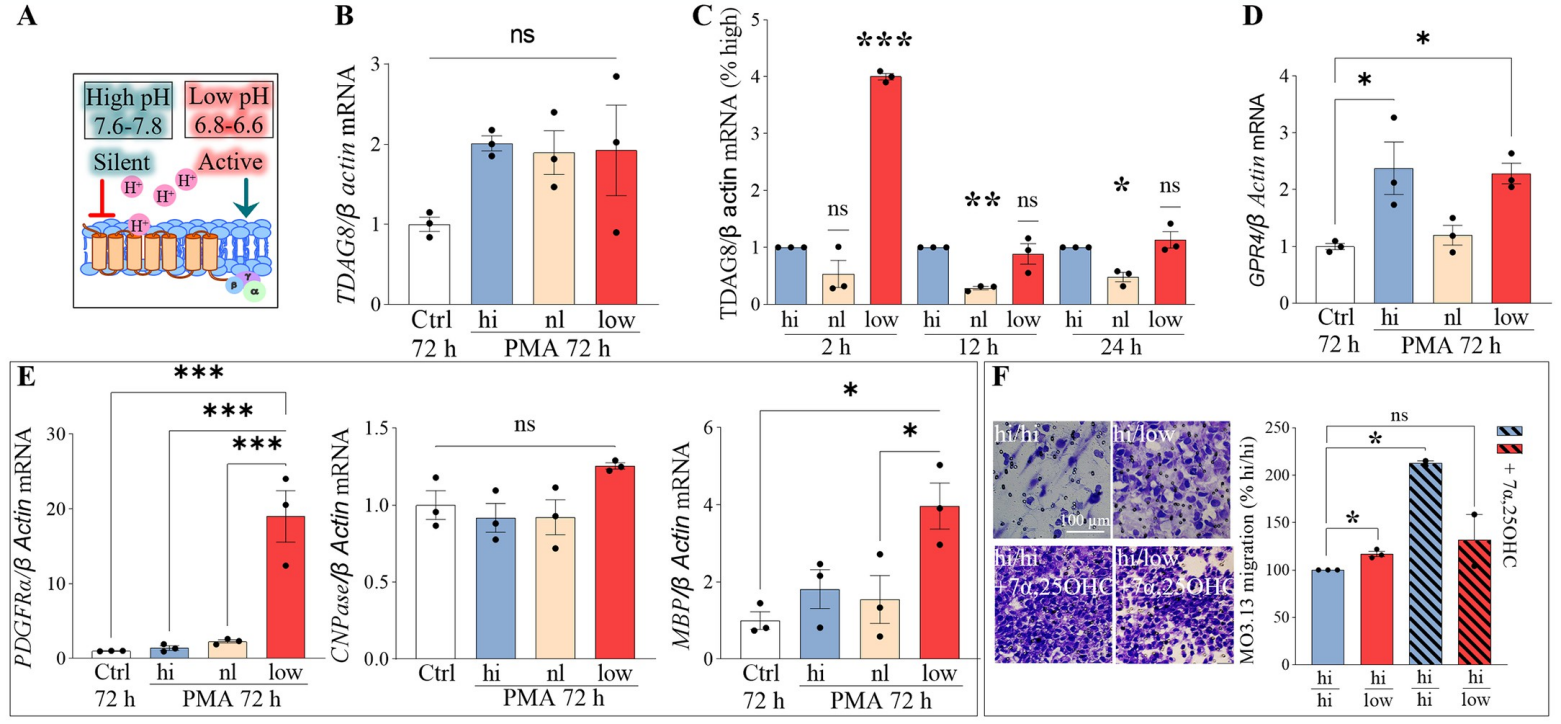
TDAG8 is upregulated in human MO3.13 oligodendrocytes during maturation and under acidic conditions. A. Schematic representation of proton-activated receptors’ signalling in different pH. B. A pH shift to either low or high pH during PMA-induced maturation of MO3.13 oligodendrocytes does not affect TDAG8 expression. One-way ANOVA and Tukey’s post-hoc tests, p>0.05, N = 3 independent experiments. C. Low pH (6.6–6.8) strongly induces TDAG8 expression after 2 h (400% +/- 6% vs. Hi) and downregulates it in normal pH after 12 h (29% +/- 3% vs. Hi) and 24 h. One sample t-test, *p≤0.05, **p≤0.01, ***p≤0.001, N = 3 independent experiments. D. A pH shift to low or high pH during human MO3.13 oligodendrocyte maturation upregulates expression of *GPR4* 2.4-fold in high and 2.3-fold in low pH. One-way ANOVA and Tukey’s post-hoc tests, p>0.05, N = 3 independent experiments. E. The *PDGFRα* mRNA transcripts in human MO3.13 oligodendrocytes were upregulated 19-fold in low pH after 72 h of differentiation with PMA. The levels of *CNPase* remained unchained and *MBP* transcripts upregulated 4-fold in low pH. One-way ANOVA and Tukey’s post-hoc tests, *p≤0.05 ***p≤0.001, N = 3 independent experiments. F. A pH shift from high to low pH stimulates the chemotaxis of MO3.13 oligodendrocytes (117% +/-2.6% vs. hi/hi). The EBI2 receptor agonist 7α,25OHC in high pH medium induces significant cell migration (212% +/- 2.5% vs. hi/hi). The 7α,25OHC-induced chemotaxis is inhibited in low pH (131.5% +/- 27.2% vs. hi/hi). One sample t-test, *p≤0.05, N = 3 independent experiments.

Our data indicated absence of TDAG8 in mouse OPCs therefore, we further explored its role in myelination and oligodendrocyte biology using the human MO3.13 oligodendrocyte cell line. Because TDAG8 is silent in high and activated in low pH, we first performed pH shift experiments during oligodendrocyte maturation (PMA treatment) to observe the effects of pH change (i.e. changes in receptor signalling) on human MO3.13 oligodendrocyte differentiation (Fig 5A). The data showed no additional pH-mediated effect on PMA-induced *TDAG8* expression at 72 hours (Fig 5B). Then, to examine if the expression of *TDAG8* is pH-dependent we performed pH shift experiments without PMA treatment. A shift to low pH, when TDAG8 is active, upregulated its expression, but only temporarily (~2 hours) and returned to baseline thereafter (~12 hours) (Fig 5C). While a shift to physiologically neutral pH significantly downregulated TDAG8 expression for at least 24 hours. *GPR4* expression was magnified with a pH change to either low or high during maturation (Fig 5D).

We also qualitatively examined the morphology of oligodendrocytes after shifting to normal (pH ~7.3), high (pH ~7.6) or low pH (~6.5). The data showed no changes in oligodendrocyte morphology when shifting to normal or high pH (silencing the receptor) (S3 Fig). However, a shift to low pH, seemed to induce mild oligodendrocyte branching. These qualitative observations should be interpreted with caution as no further analysis was performed. Similarly, no qualitative differences in the level of PDFRGα, CNPase or A2B5 in oligodendrocytes were observed after 24 h culture in either normal, high or low pH. Subsequent analysis of the oligodendrocyte differentiation markers at mRNA level revealed that long-term cell culture in low pH induces expression of the OPC marker *PDGFRα* and, surprisingly, *MBP* and has no effect on *CNPase* mRNA levels (Fig 5E) [27].

Lastly, in light of our findings that acidic pH-induced *TDAG8* expression and sustained the expression of the early OPC marker PDGFRα, we investigated the impact of TDAG8 activation (induced by a shift to acidic pH) on the migratory behaviour of undifferentiated MO3.13 oligodendrocytes. OPC motility is vital for their function, particularly in the context of remyelination—where new myelin is formed either by the surviving adult oligodendrocytes or by OPCs recruited to injury sites in response to various chemoattractants and acidic pH [40]. The data showed that a pH shift from high (silenced receptor) to low (receptor active) had a minor chemotaxis-inducing effect on oligodendrocytes compared to no pH shift (maintenance in high pH) (Fig 5F). Subsequently, to compare the level of migration induced by low pH to a known chemotaxis-inducing agent in MO3.13 oligodendrocytes [27, 41], we stimulated the cells with EBI2 receptor agonist, oxysterol 7α,25OHC, in either low or high pH. The MO3.13 oligodendrocytes extensively migrated towards 7α,25OHC in high pH. Strikingly, the 7α,25OHC-induced chemotaxis was significantly reduced in low pH indicating that an acidic environment inhibits oligodendrocyte migration induced by other than pH chemotactic agents.

## Discussion

TDAG8 has been identified as a risk gene for MS in GWAS studies [13, 14]. In our investigation, we observed a significant upregulation of TDAG8 in MS lesions. Importantly, neither GPR4 nor OGR1 showed deregulation in MS brains. This emphasizes the potential role of the TDAG8 receptor in the pathogenesis of MS. It’s crucial to highlight that the elevated TDAG8 transcripts in the white matter plaques may stem from increased infiltration by reactive lymphocytes, which not only express TDAG8 but are also concentrated in MS plaques [42]. Supporting this notion, an RNA-seq study revealed a decreased expression of TDAG8 in the corpus callosum and the optic chiasm of MS patients [43], indirectly reinforcing our conclusion regarding the source of increased TDAG8 expression in the plaques as the infiltrating lymphocytes. However, further investigation is needed to determine whether TDAG8 is also upregulated in the central nervous system (CNS) and immune-competent resident cells, such as microglia or astrocytes, in the MS brain. This exploration could potentially uncover new opportunities for modulating glial reactivity and inflammatory signalling in MS.

Using the cuprizone model of MS model and RNA-seq, a large study demonstrated that *tdag8* is strongly downregulated during the remyelination phase [43]. Here, we demyelinated organotypic cerebellar slices with LPC and examined gene expression during spontaneous remyelination. In line with our findings of no TDAG8 expression in mouse oligodendrocytes, only *gpr4* transcripts were downregulated. Both studies implicate the potential involvement of the pH-sensing receptors in remyelination but the precise mechanisms need to be pinpointed as they clearly vary between the species, and applied models. Notably, the data underscores the identified species differences in the pH-sensing receptor function during neuroinflammation and remyelination with GPR4 playing a more prominent function in the mouse brain and oligodendrocytes and TDAG8 in the human cells. Interestingly, we found that the expression of OGR1 and GPR4 are affected in TDAG8 KO mice brains. These observations agree with the apparent opposing roles of the anti-inflammatory TDAG8 and pro-inflammatory GPR4 [44]. In the absence of TDAG8, the CNS expression levels of OGR1 and GPR4 are upregulated, thus changing the balance towards pro-inflammatory signalling. Also during remyelination in cerebellar slices, the expression of GPR4 was downregulated suggesting an anti-inflammatory profile in this tissue during remyelination.

The pH-sensing receptors, TDAG8 and GPR4, are mainly expressed in the immune cells and modulate inflammatory signalling in various diseases including autoimmune diseases of the CNS such as MS (reviewed in [38]). We, therefore, investigated whether an inflammatory challenge of either cells *in vitro* or mice *in vivo* with different pro-inflammatory agents affects receptor expression in the brain. Previous studies showed that a combined treatment of cells *in vitro* with TNFα and IL17 induces an extensive release of pro-inflammatory cytokines including IL6 involving the NFκB signalling pathway [39, 45]. Importantly, the release of the pro-inflammatory mediators is greater when IL17 and TNFα are combined than when either is given alone. Specifically in oligodendrocytes, treatment with IL17 was shown to synergistically augment the TNFα-induced oligodendrocyte apoptosis, mitochondrial dysfunction, release of reactive oxygen species and induce an arrest of OPC maturation, thus promoting myelin damage [45]. In our experiments, the co-treatment of the human MO3.13 oligodendrocytes induced an overall downregulation of *TDAG8* transcripts irrespective of the extracellular pH but most pronounced in low and high pH respectively implicating that the anti-inflammatory signalling through TDAG8 receptor may be directly affected by the cytokines mediating MS pathogenesis [46–50]. However, the non-pH-specific reaction to TNFα/IL17 renders it difficult to draw any conclusions regarding the possible role of TDAG8 in the TNFα/IL17-mediated oligodendrocyte dysfunction and should be dissected in future studies using receptor ligands or TDAG8 KO animals. A comprehensive investigation could deliver a novel mechanism for limiting oligodendrocyte loss in demyelinating diseases such as MS.

The Increased TDAG8 expression reported here after LPS challenge in cultured human MO3.13 oligodendrocytes and the mouse brain is in line with the previous reports of the anti-inflammatory role of TDAG8 in LPS-stimulates peritoneal macrophages and T cells [51, 52]. In these studies activation of TDAG8 in acidic pH reduced the levels of pro-inflammatory cytokines (TNFα, IL6) and increased the synthesis of the anti-inflammatory IL10 cytokine after LPS challenge. In the LPS-induced acute lung injury model, the expression of TDAG8 was also induced in the lungs and local macrophages, and the lung damage was increased in TDAG8-deficient mice, again indicating the anti-inflammatory role of TDAG8 [53]. Our investigations indicate that TDAG8 is involved in inflammatory signalling in the brain upon peripheral bacterial infection and should be further elucidated to answer such questions as to whether modulation of its signalling may be used to attenuate neuroinflammation and associated cell injury and demyelination.

Local changes in extracellular pH, which accompany inflammation and tissue injury, should attract local and peripheral immune cells, as well as OPCs, to the affected area to effectively prepare the milieu for regeneration and remyelination. Our investigations indicated that a shift from high to low pH moderately induced oligodendrocyte chemotaxis and inhibited 7α,25OHC-induced migration indicating that low pH could have an overall inhibitory effect on oligodendrocyte chemotaxis. Similar observations were made by Jagielska and colleagues (2013) who found that at a fixed low pH of 6.0, the migration velocity and radius of primary rat OPCs are lower compared to the pH ranges of 6.5 and higher (pH 6.8 in our experiments). However, when a pH gradient was formed in Zigmond chambers the oligodendrocytes migrated towards the acidic pH (pH 6 versus pH 7) indicating that OPCs may indeed be attracted to the lesion or inflammatory site where a pH gradient forms, with more acidic pH closer to the wound. The adhesion and length of OPCs increased in acidic pH, in line with the decreased migration reported in the low pH experiments. Moreover, the rat OPC viability, proliferation and differentiation were significantly decreased in the low pH of 6.0–6.5 compared to the neutral pH of 7.0–7.5. Neither of the previous research discussed here, including ours, used receptor-specific ligands rendering it impossible to conclude whether TDAG8 or GPR4 are involved in oligodendrocyte migration in acidic pH. In light of the lack of well-characterised, potent and selective TDAG8 ligands and only an antagonist (NE 52-QQ57) for GPR4, future investigations should use GPR4- and TDAG8-deficient oligodendrocytes to decipher whether these receptors modulate oligodendrocyte migration under inflammatory and acidic conditions.

We attempted to examine some aspects of oligodendrocyte differentiation/maturation. Our investigations revealed increasing transcripts of *TDAG8* in human MO3.13 oligodendrocytes and *gpr4* in primary mouse OPCs. The expression of TDAG8 was further upregulated in low pH but only temporarily. Interestingly, only when combined, the low pH and the differentiating factor PMA sustained the expression of the OPC marker PDGFRα and induced the expression of mature oligodendrocyte marker MBP suggesting that TDAG8 may be involved in OPC/pre-oligodendrocyte function related to myelination under pathophysiological conditions (low pH). Indeed, superficial examination of the cell morphology revealed elongation of the cell processes only in the acidic pH. A previous study using primary rat oligodendrocytes also found that acidic pH induces elongation of cell processes but inhibits cell differentiation, as indicated by low MBP levels [40]. Bernard and colleagues [54], on the other hand, observed the highest differentiation of primary rat cerebellar oligodendrocytes at pH 7.15 and a declining rate of differentiation (GalC levels) at either lower or higher pH ranges. None of the previous studies examined the OPC marker PDGFRα, which was strongly upregulated (or sustained) in our study at low pH. Further investigation is required to determine whether acidic pH indeed inhibits oligodendrocyte maturation and/or maintains OPCs in an undifferentiated state marked by PDGFRα expression. Additionally, it remains to be explored whether TDAG8 or GPR4 play a role in these processes. Subsequent studies should employ diverse in vitro models to scrutinize these phenomena. The hybrid human-human adult oligodendrocyte MO3.13 cell line, however, is not well-suited for studying OPC differentiation and maturation. Although primary rodent OPCs are easily obtainable, our investigations revealed the absence of TDAG8 in mouse cells, making this model unsuitable for studying these functions of TDAG8. Given the scarcity and difficulty in obtaining human primary OPCs, the use of human iPSC-derived OPCs and brain organoids, which contain OPCs and oligodendrocytes at various stages of maturation and differentiation, appears to be the most optimal approach in these studies.

A significant constraint and limitation in our study arises from the lack of well-defined pharmacological tools, such as specific agonists and antagonists, especially for TDAG8. Lowering the extracellular pH does not necessarily translate to the activation of these receptors. Various other proteins and pH-sensing receptors are affected by alterations in extracellular pH, including OGR1, acid-sensing ion channels, GPR132, as well as transient receptor potential vanilloids 1 and 4. Moreover, low pH could also affect other non-pH sensing receptors that have been shown to regulate OPC differentiation and migration, such as EBI2 or the mechanosensing ion channel Piezo1, which is inhibited in pH under 6.9 [27, 28, 55, 56]. Nevertheless, looking at the myelination state *in vivo* in TDAG8 KO mice, our explorations demonstrate that TDAG8-mediated signalling is not required for normal myelination in the mouse CNS.

### Conclusions

The identification of species-specific differences in proton-sensing receptor expression within the oligodendrocyte lineage carries significant implications for drug development, the interpretation of data derived from mouse models, and the challenges inherent in translating findings from preclinical models to clinical studies. While TDAG8 may play pertinent roles in myelination processes, the mouse emerges as a suboptimal species for studying its involvement. Therefore, despite overarching observations, our study does not furnish robust evidence for the role of TDAG8 in such processes. However, it is imperative to underscore that this manuscript significantly contributes to the body of knowledge, even with predominantly negative results. By illuminating crucial species differences in pH-sensing receptor expression in the brain, our findings have far-reaching implications. Acknowledging the limitations of the available models the study provides valuable insights for directing future efforts in dissecting the role of TDAG8 in myelination and MS. It also emphasizes the necessity of employing appropriate models for studying these intricate processes.

## Supporting information

S1 Table The list of primary antibodies used for immunofluorescent stainings. (TIF)

S2 Table The list of FAM-labelled TaqMan Gene Expression probes used in this study. (TIF)

S1 Fig A. Treatment of MO3.13 oligodendrocytes *in vitro* with a cocktail of TNFα/IL17 pro-inflammatory cytokines downregulates *TDAG8* expression by 80% in high pH and 68% in low pH. One sample t-test, *p≤0.05, **p≤0.01, N = 3 independent experiments. B. A challenge with bacterial LPS (100 ng/ml) induces *TDAG8* expression 3-fold after 2 hours and 3.4-fold after 24 hours. One sample t-test, *p≤0.05, N = 3 independent experiments.
(TIF)

S2 Fig A. Treatment of MO3.13 oligodendrocytes *in vitro* with a cocktail of TNFα/IL17 pro-inflammatory cytokines downregulates *TDAG8* expression by 80% in high pH and 68% in low pH. One sample t-test, *p≤0.05, **p≤0.01, N = 3 independent experiments. B. A challenge with bacterial LPS (100 ng/ml) induces *TDAG8* expression 3-fold after 2 hours and 3.4-fold after 24 hours. One sample t-test, *p≤0.05, N = 3 independent experiments.
(TIF)

S3 Fig Representative images showing human MO3.13 oligodendrocytes stained with anti-PDGFRα (green), anti-CNPase (green) and anti-A2B5 (red) antibodies after 24-hour culture in either normal, high or low pH. The low pH-induced elongation of cell processes (arrows). Normal pH = 7.2; high pH = 7.6, low pH = 6.8.
(TIF)

S4 Fig *TDAG8* is expressed in human OPCs and microglia. (TIF)
