# Supplementary figures and images for: The proton-sensing receptors TDAG8 and GPR4 are differentially expressed in human and mouse oligodendrocytes: Exploring their role in neuroinflammation and multiple sclerosis
Source: PLoS One. 2024 Mar 25;19(3):e0283060. doi: 10.1371/journal.pone.0283060 (PMC10962805; doi:10.1371/journal.pone.0283060)

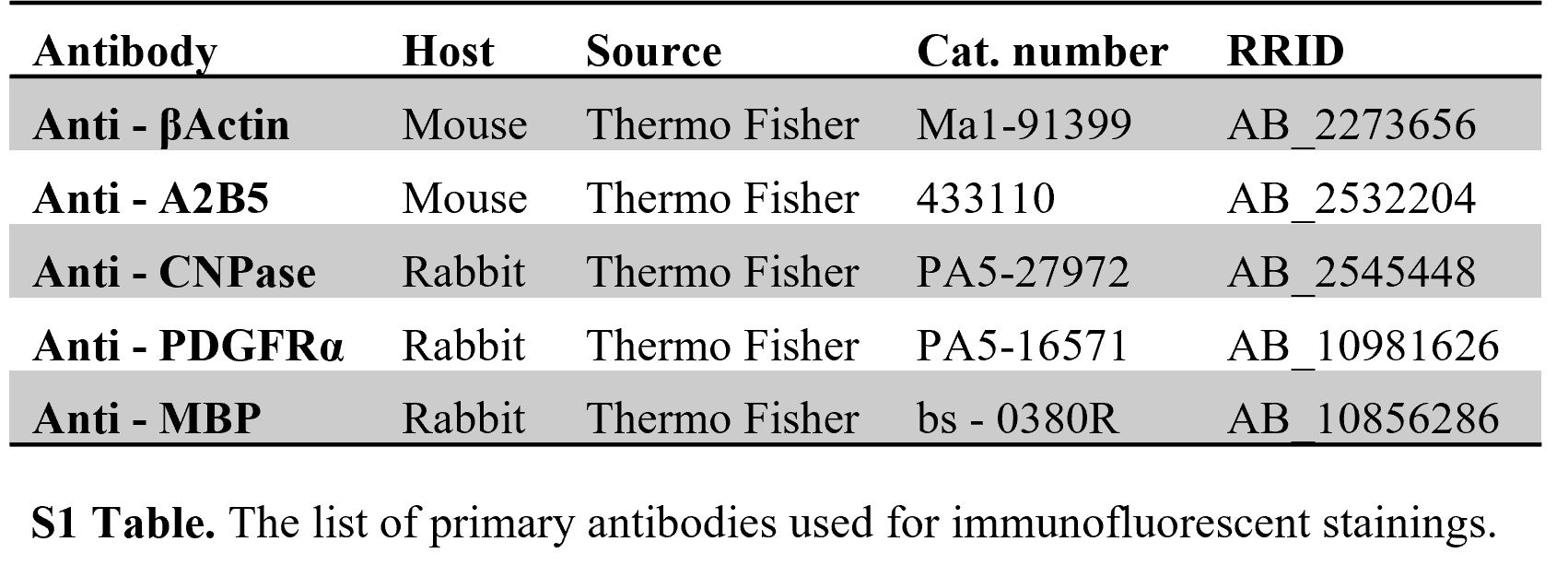

Supplement: S1 Table — (TIF) [file pone.0283060.s001.tif]

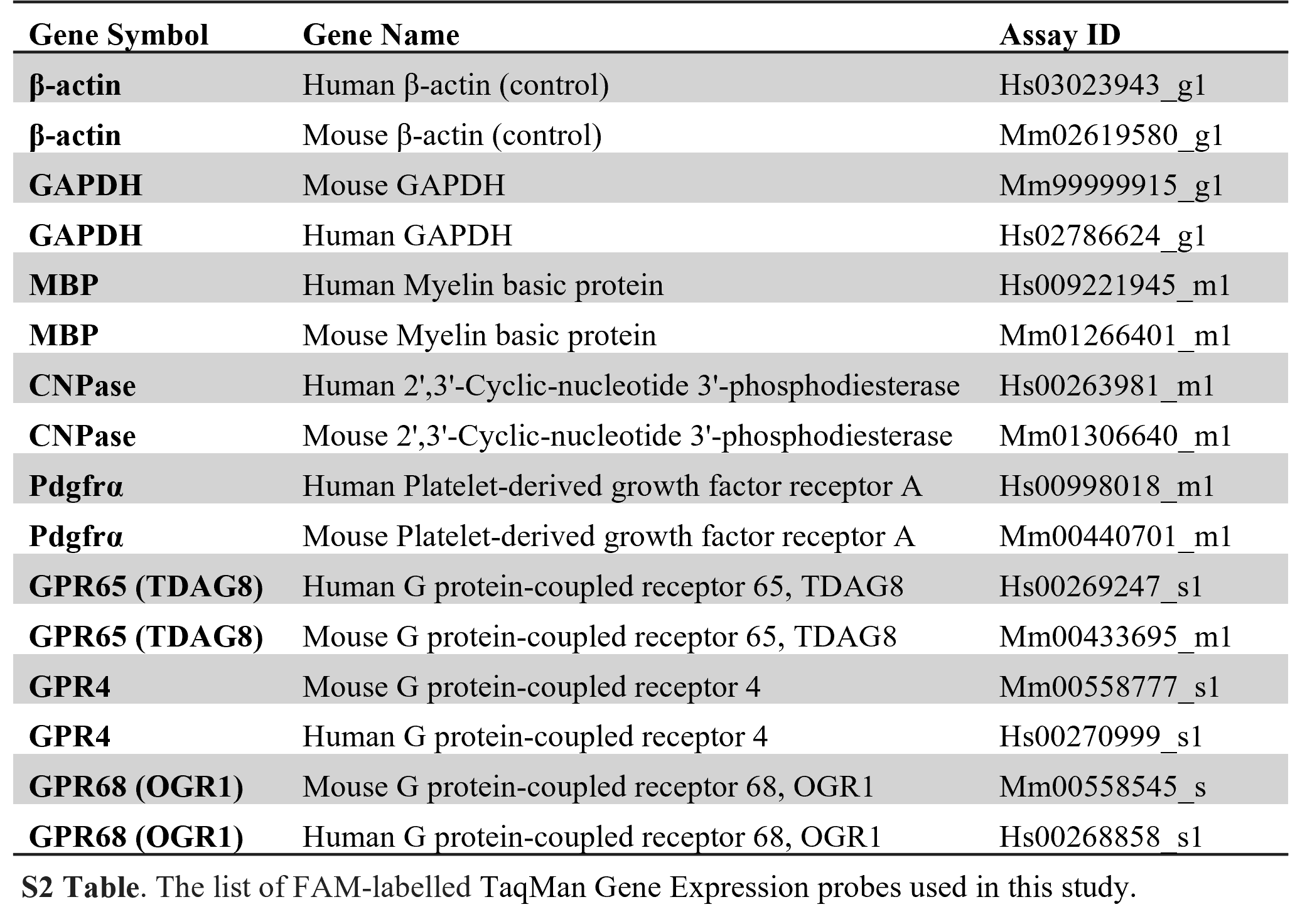

Supplement: S2 Table — (TIF) [file pone.0283060.s002.tif]

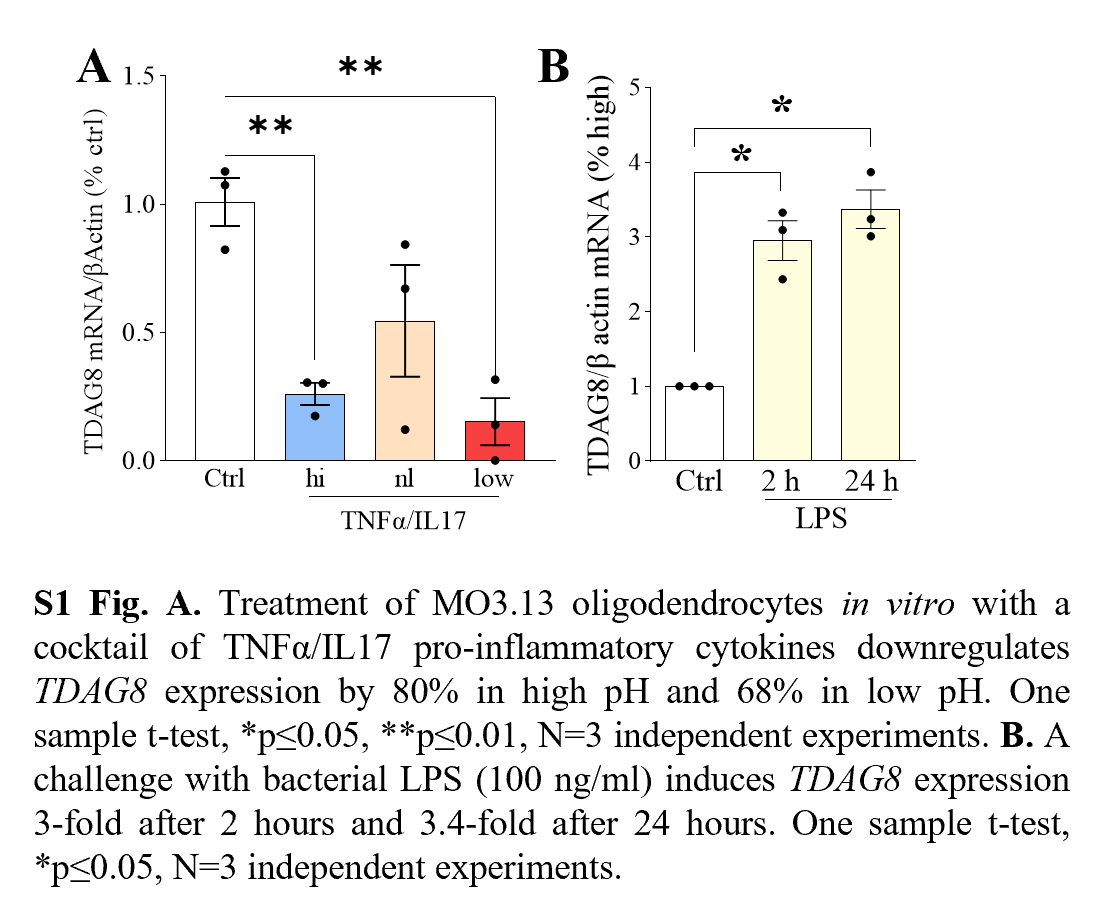

Supplement: S1 Fig — A. Treatment of MO3.13 oligodendrocytes in vitro with a cocktail of TNFα/IL17 pro-inflammatory cytokines downregulates TDAG8 expression by 80% in high pH and 68% in low pH. One sample t-test, *p≤0.05, **p≤0.01, N = 3 independent experiments. B. A challenge with bacterial LPS (100 ng/ml) induces TDAG8 expression 3-fold after 2 hours and 3.4-fold after 24 hours. One sample t-test, *p≤0.05, N = 3 independent experiments. (TIF) [file pone.0283060.s003.tif]

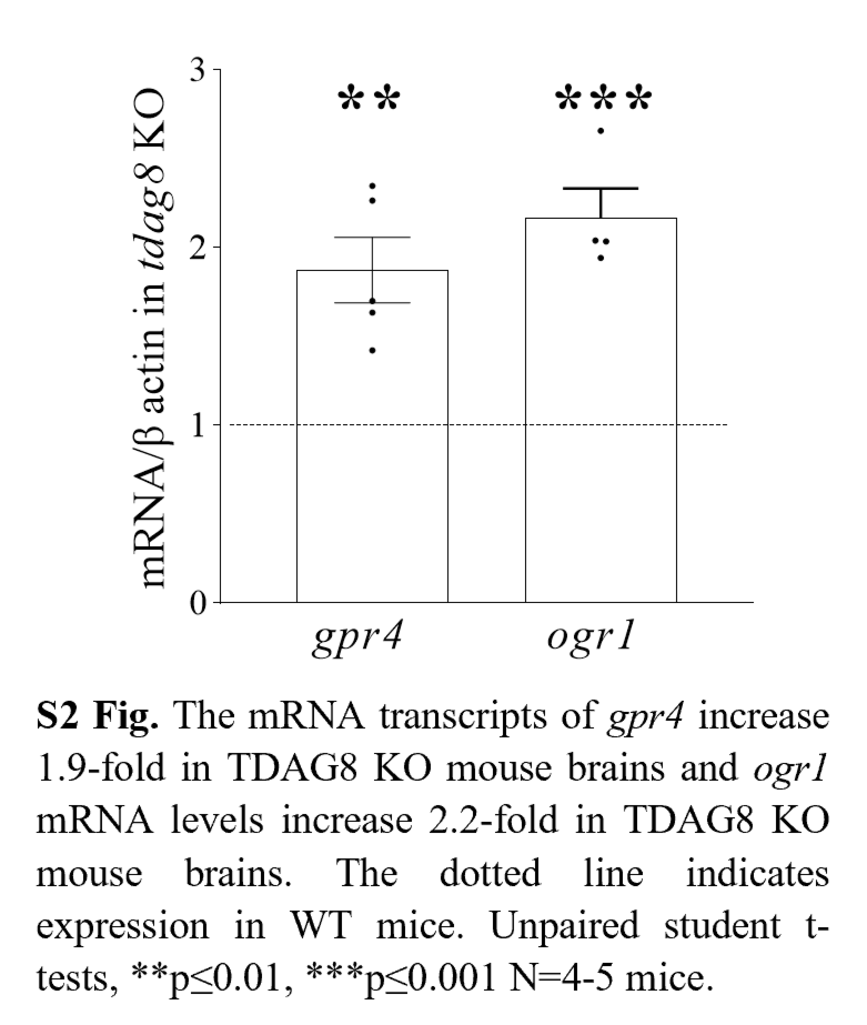

Supplement: S2 Fig — A. Treatment of MO3.13 oligodendrocytes in vitro with a cocktail of TNFα/IL17 pro-inflammatory cytokines downregulates TDAG8 expression by 80% in high pH and 68% in low pH. One sample t-test, *p≤0.05, **p≤0.01, N = 3 independent experiments. B. A challenge with bacterial LPS (100 ng/ml) induces TDAG8 expression 3-fold after 2 hours and 3.4-fold after 24 hours. One sample t-test, *p≤0.05, N = 3 independent experiments. (TIF) [file pone.0283060.s004.tif]

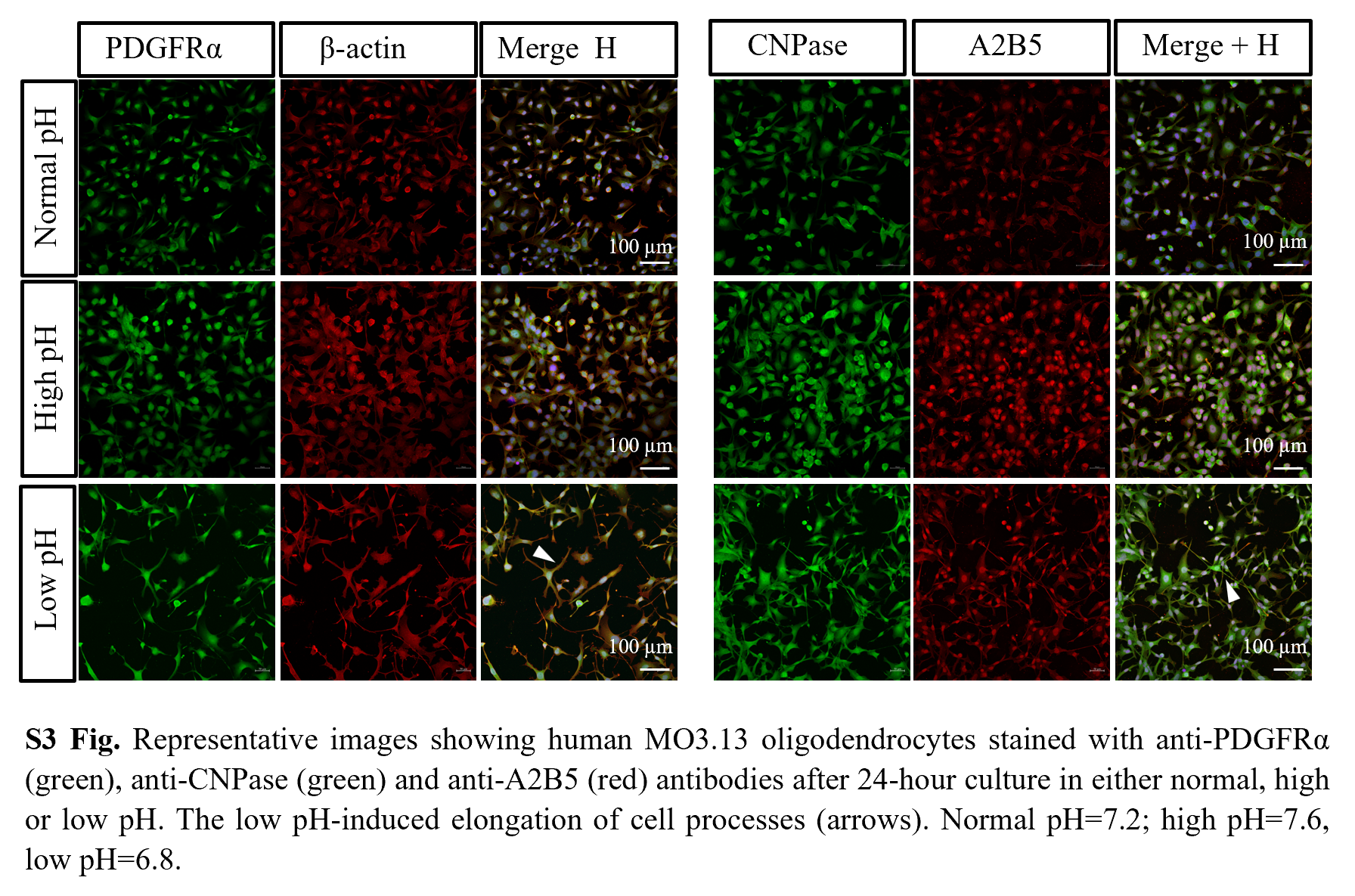

Supplement: S3 Fig — The low pH-induced elongation of cell processes (arrows). Normal pH = 7.2; high pH = 7.6, low pH = 6.8. (TIF) [file pone.0283060.s005.tif]

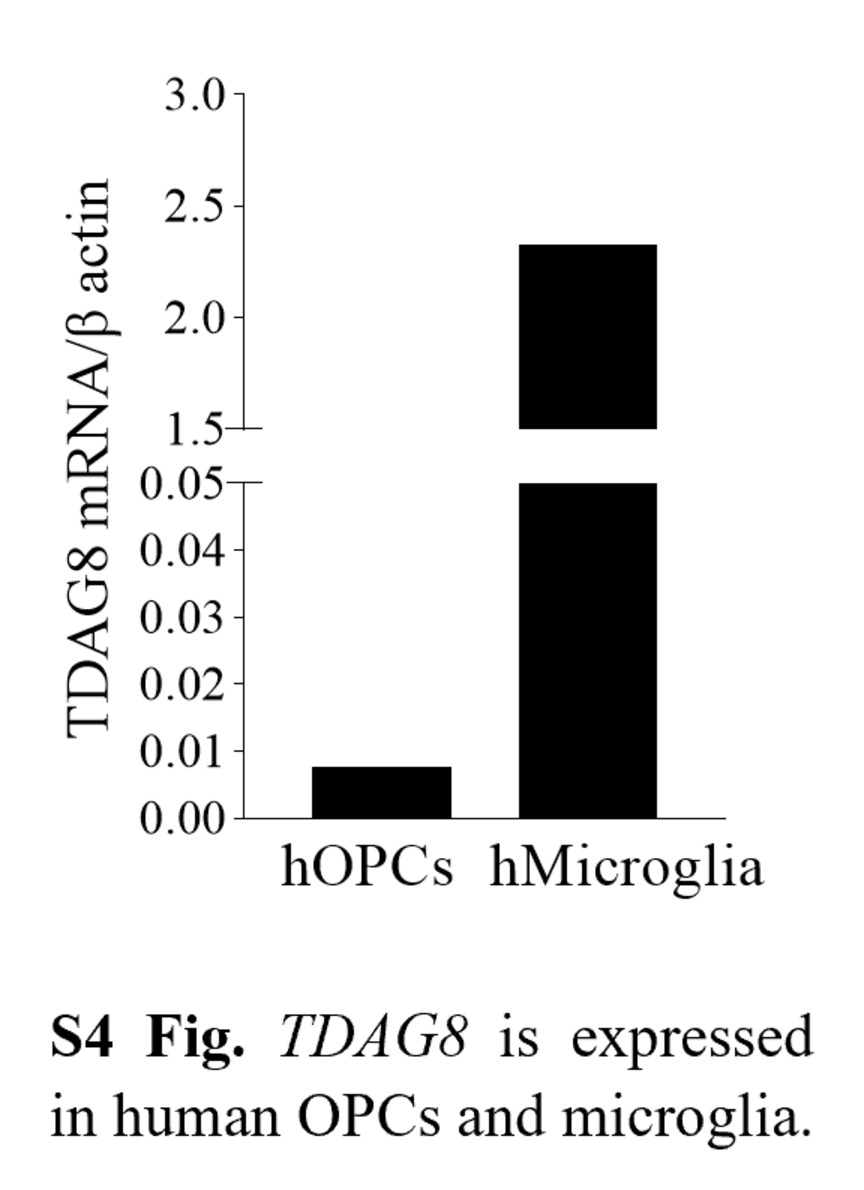

Supplement: S4 Fig — (TIF) [file pone.0283060.s006.tif]
